# CFC1 is a cancer stemness-regulating factor in neuroblastoma

**DOI:** 10.18632/oncotarget.18464

**Published:** 2017-06-13

**Authors:** Koji Chikaraishi, Hisanori Takenobu, Ryuichi P. Sugino, Kyosuke Mukae, Jesmin Akter, Masayuki Haruta, Masafumi Kurosumi, Takaho A. Endo, Haruhiko Koseki, Naoki Shimojo, Miki Ohira, Takehiko Kamijo

**Affiliations:** ^1^ Research Institute for Clinical Oncology, Saitama Cancer Center, Saitama, Japan; ^2^ Department of Pediatrics, Chiba University, Chiba, Japan; ^3^ Laboratory of Tumor Molecular Biology, Graduate School of Science and Engineering, Saitama University, Saitama, Japan; ^4^ Department of Pathology, Saitama Cancer Center, Saitama, Japan; ^5^ Laboratory for Integrative Genomics, RIKEN Center for Integrative Medical Sciences, Kanagawa, Japan; ^6^ Laboratory for Developmental Genetics, RIKEN Center for Integrative Medical Sciences, Kanagawa, Japan

**Keywords:** CFC1, neuroblastoma, cancer stem cells, sphere, Activin A

## Abstract

**Background:**

Despite the use of aggressive therapy, survival rates among high-risk neuroblastoma (NB) patients remain poor. Cancer stem cells (CSCs) are considered to be critically involved in the recurrence and metastasis of NB and are isolated as NB spheres.

**Methods:**

The gene expression profiling of adherent (control) and sphere-forming primary NB cells was conducted using a gene expression microarray. CFC1, which functions in the development of embryos and decides the left-right axis, was strongly expressed in sphere-forming cells only and was related to the unfavorable prognosis of NB patients. The knockdown and overexpression of *CFC1* were performed using a lentiviral system in NB cell lines. Sphere formation, cell proliferation, colony formation in soft agar, and xenograft tumor formation were analyzed.

**Results:**

The overexpression of CFC1 increased sphere formation, cell growth, and colony formation. These phenotypes, particularly sphere formation, and xenograft tumor formation were significantly suppressed by the knockdown of *CFC1*. CFC1 inhibited Activin A-induced NB cell differentiation and Smad2 phosphorylation in NB cell lines, indicating its involvement in tumorigenesis related to EGF-CFC co-receptor family molecule pathways. Collectively, these results indicate that CFC1 is a candidate molecule for the development of CSC-targeted therapy for NB.

## INTRODUCTION

Accumulating evidence has shown that cancer stem cells (CSCs) are a small subpopulation of tumor cells that exhibit the properties of self-renewal, differentiation into various tumor cell types, tumorigenicity, and chemo/irradiation resistance [1]. Due to their self-renewal property and strong tumorigenic ability, CSCs are regarded as the starting point for tumors and are considered to be critically involved in relapse and metastasis [2]. Therefore, CSCs have become promising target cancer cells for preventing cancer relapse and improving the prognosis of tumor patients [3].

The identification of CSCs was achieved by isolating highly tumorigenic tumor cells from primary tumors and tumor cell lines. In the isolation of highly tumorigenic cells, antibodies against CSC markers have been key in the progression of CSC research. Cells positive for the membrane protein CD133 were initially regarded as CSCs in various types of cancers including brain tumors, colon cancers, hepatomas, and gastric cancer [4, 5]; however, CD133-negative cells have also been shown to initiate tumors [6]. In breast cancer, CD44+/CD24−/low cells and ALDH-positive cells exhibit stem cell features, and a large number of studies have confirmed the poor prognosis of tumors with these CSC markers [7, 8]. These findings demonstrated that the characterization of CSCs may be based not only on cell surface markers, but also on other robust properties such as the tumorigenic and self-renewal abilities of tumor cells.

A tumor sphere is a solid, spherical formation that develops from the proliferation of cancer stem/progenitor cells. These tumor spheres are easily distinguishable from single or aggregated cells because they appear to fuse together and individual cells cannot be identified. Cells are grown under serum-free, non-adherent conditions and the cancer stem/progenitor cell population is enriched when cancer stem/progenitor cells survive and proliferate in this environment [9, 10]. Only approximately 50% of high-grade tumors are available for continuous cultivation as tumor spheres [11].

Neuroblastoma (NB) is the most common pediatric extracranial solid tumor and is derived from sympathetic neuron-related tissues [12]. Advanced stage tumors and *MYCN*-amplified tumors show high percentages of relapse, even after the application of combined multimodal therapies that are currently available [13], suggesting the presence of cancer stem NB cells in these aggressive NB tumors. CSCs in NB were found to be enriched by sphere formation in sphere-forming medium (SFM) containing bFGF and EGF, which are the conditions used for neural crest stem cell growth [14, 15]. Highly tumorigenic NB CSC-candidate cells were successfully enriched by non-serum/bFGF/EGF medium because as few as 10 passaged tumor sphere cells from aggressive NB injected orthotopically into severe combined immune-deficient/Beige mice formed large NB tumors that metastasized to the liver, spleen, contralateral adrenal gland and kidney, and lungs [14]. Serum-free medium containing bFGF and EGF successfully enriches NB tumor-initiating cells (Akita et al., manuscript in preparation) and is useful for examining the functions of the stemness-related CSC marker CD133 in NB [16].

The development of new therapies that target NB CSCs may be useful for preventing or treating tumor recurrence. Therefore, we used a human exon microarray to profile the transcriptomes of 2 NB tumor spheres from 2 NB patients and then selected candidate molecules using a survival analysis, UniProt-GOA database information, and an RT-PCR analysis of sphere-forming NB cell lines. CFC1, a member of the epidermal growth factor-Cripto/FRL-1/Cryptic (EGF-CFC) family, was identified as an NB stemness-related molecule. Lentivirus-mediated biological *in vitro/in vivo* experiments confirmed the significance of CFC1 in NB stemness and the molecular mechanisms underlying CFC1-induced phenomena were investigated.

## RESULTS

### Cancer stemness-related molecules in NB

**Figure 1:**
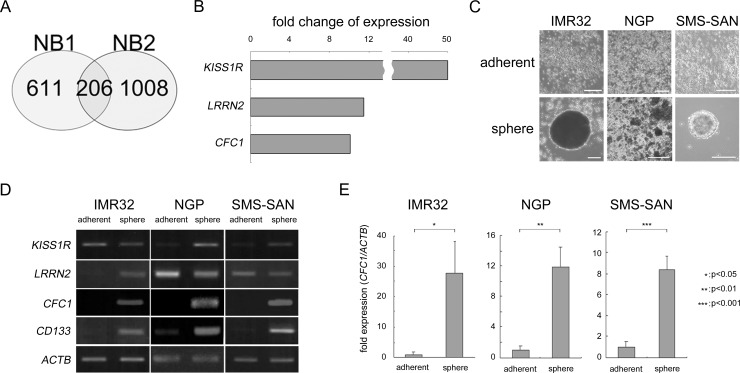
Target molecule selection by the cancer stem cell model in NB **A**. Venn diagram of the overlap among up-regulated genes in NB1 and NB2 tumor spheres. Genes up-regulated by more than 2-fold in spheres from tumor tissues and related to an unfavorable prognosis were selected using microarrays and a survival data analysis. **B**. Fold changes in three genes ranked in the Top 15 functioning as cell surface receptors or co-receptors (Supplementary Table 2). **C**. NB cell lines were cultured in six-well culture plates with normal (adherent) or sphere-forming medium for 3 weeks (sphere). Bar, 50 μm. **D**. Semi-quantitative RT-PCR was performed using specific primers against *KISS1R*, *LRRN2*, *CFC1*, and *CD133*. *ACTB* was used as a loading control. **E**. Quantitative PCR (qPCR) analysis of *CFC1*. Relative *CFC1* values were normalized by *ACTB* values. Data are representative results of three independent experiments. A statistical analysis was performed using the Student's *t-*test.

In order to elucidate the mechanisms responsible for NB cancer stemness, we examined tumor spheres as a model of NB CSCs. Two primary NB cells from patients (NB1 and NB2) were cultured using the sphere-forming culture method described in MATERIALS AND METHODS. They were derived from the bone marrow of two Stage 4 patients and were free from EB virus infection (Supplementary Figure 1). We then searched for NB stemness-related genes. NB stemness-related genes are assumed to have two characteristics: gene expression levels are high in tumor spheres and strong expression is an indicator of a poor prognosis. Gene expression levels were measured using an Affymetrix microarray (Affymetrix GeneChip^®^ Human Genome U133 Plus 2.0 Array). A change in gene expression was defined as a more than 2-fold change in expression levels. The survival effect of gene expression in neuroblastoma patients was previously summarized [17]. “Either high or low is worse” for each gene was defined by the R2 “scan” algorithm (R2: Genomics Analysis and Visualization Platform (http://r2.amc.nl), see MATERIALS AND METHODS for details). After these analyses, 817 and 1214 genes were selected in NB1 and NB2 samples, respectively. In a Venn diagram created using these data, 206 genes were commonly observed in NB1 and NB2 spheres (Figure 1A). We ranked these genes according to fold changes and the top 15 genes were listed in Supplementary Table 1. We also examined their subcellular localization by UniProt-GOA (www.ebi.ac.jk/GOA). Three molecules belonging to cell surface receptors or co-receptors were selected (Figure 1B). We then produced tumor spheres using NB cell lines. IMR32, NGP, and SMS-SAN cells efficiently produced tumor spheres under SFM conditions (Figure 1C). RNA expression levels were measured in these tumor spheres by RT-PCR using specific primers against *KISS1R*, *LRRN2*, and *CFC1*. *ACTB* was used as a loading control. *CD133* was previously reported to be a specific marker of NB spheres [16]. The expression of *CFC1* and *CD133* was markedly up-regulated in tumor spheres (Figure 1D). The EGF-CFC family molecule TDGF1 (Cripto, CR-1) plays important roles in the tumorigenesis and aggressiveness of many cancers [18]. Based on the up-regulation of expression by sphere formation, subcellular localization, and molecular functions, we selected CFC1 as a candidate stemness-related molecule in the present study. In the quantitative RT-PCR (qPCR) analysis, *CFC1* expression normalized by *ACTB* was also strongly up-regulated in the tumor spheres of all three cell lines (Figure 1E).

EGF-CFC family members function as co-receptors for TGFβ family signals. Ligands such as TGFβ, Nodal, and Activin A bind to Activin Receptor 2A (ACVR2A) or ACVR2B with ACVR1B. The activation of these receptors stimulates Smad2 phosphorylation and downstream signals [19]. In order to study the role of EGF-CFC family molecules in NB tumor sphere formation, the expression levels of *CFC1*, *TDGF1*, *ACVR2A*, *ACVR2B*, and *ACVR1B* were measured using a microarray. Apart from *CFC1*, these genes were not up-regulated in sphere-forming NB1 or NB2 cells. The stemness markers *SALL4* and *NANOG* were strongly expressed in tumor spheres (Supplementary Figure 2). We also reviewed their effects on patient prognoses using an R2 database Kaplan-Meier analysis (Supplementary Table 2, Supplementary Figure 3). High expression levels of *CFC1* strongly correlated with an unfavorable prognosis. We also detected CFC1 in NB1 sphere-forming NB cells using a FACS analysis (Supplementary Figure 4), which suggested that only a small population of sphere-forming NB cells expressed CFC1.

The relationship between the expression of *CFC1* and *MYCN* was examined using the R2 database. The strong expression of CFC1 correlated with a poor prognosis in patients in whom *MYCN* was not amplified. However, the prognosis of patients with amplified *MYCN* was poor regardless of the expression of *CFC1*. Furthermore, CFC1 expression levels were not significantly changed by the MYCN status of patients (Supplementary Figure 5).

### *CFC1* depletion suppresses NB cell aggressiveness

**Figure 2:**
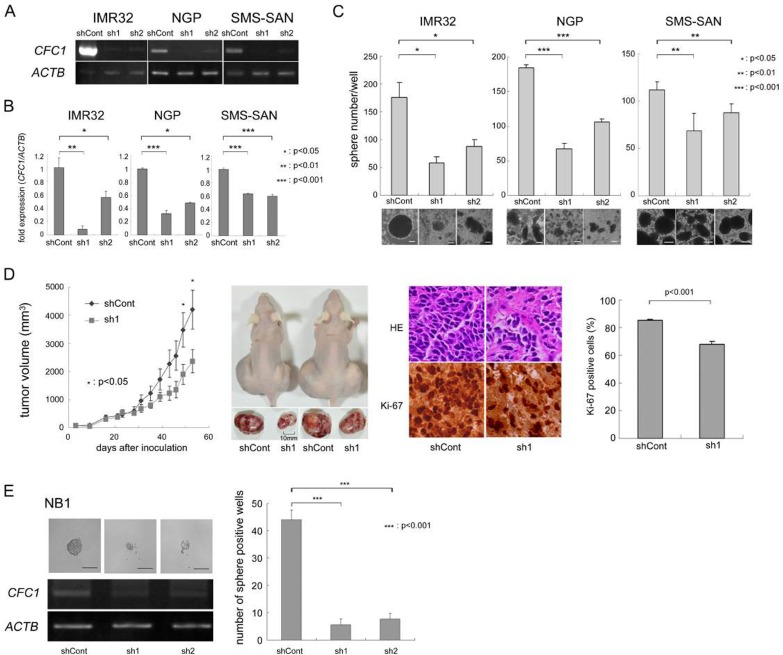
CFC1 depletion suppresses tumor sphere and xenograft formation **A**. Stable knockdown of *CFC1* by lentivirus-mediated shRNA. *CFC1* was knocked down by shRNAs in IMR32, NGP, and SMS-SAN cells. *CFC1* expression was analyzed in NB tumor spheres by RT-PCR. **B**. qPCR was performed on *CFC1* and normalized by *ACTB*. **C**. Sphere numbers were counted after a 3-week SFM culture. **D**. Tumor development in BALB/c AJcl nu/nu mice following the injection of IMR32 cells stably infected with shRNA against the control (shCont) and *CFC1* (sh1). Tumor volumes were measured every 2 or 3 days. Data are presented as the mean ± SE of tumors in four mice. Immunohistochemistry was performed using hematoxylin-eosin and Ki-67. The % of Ki-67-positive cells was counted. Data are presented as the percentages of Ki-67-positive cells/field (mean ± SD) in at least three independent fields. The original magnification of each panel is ×400. **E**. *CFC1* knockdown was performed on the primary NB sphere, NB1. *CFC*1 shRNA was infected into NB1 spheres as described in the MATERIALS AND METHODS. Two days after infection, spheres were loosened with an equal volume of AccuMax^®^ and seeded at 250 cells per each well on a 96-well plate with 100 μL SFM. The number of sphere-positive wells was counted 4 days after infection. All data in this figure are representative results of at least three independent experiments and all statistical analyses were performed using the Student's *t*-test.

We knocked down *CFC1* in three NB cell lines (IMR32, NGP, and SMS-SAN) using two types of shRNA (sh1 and sh2) in the lentiviral system described in MATERIALS AND METHODS. Control shRNA (shCont) was simultaneously infected and used as a negative control. After three weeks in the SFM culture, we assessed the expression of *CFC1* using RT-PCR (Figure 2A) and qPCR (Figure 2B). In *CFC1* knocked down cells, sphere-forming ability was significantly decreased in all three cell lines (Figure 2C). We performed a WST assay and soft agar colony assay and found that the knockdown of *CFC1* did not influence cell proliferation under normal or anchorage-independent conditions (data not shown). In order to investigate tumorigenicity *in vivo*, *CFC1*-depleted IMR32 cells were injected subcutaneously into the backs of nude mice. *CFC1* shRNA-infected cells (sh1) formed significantly smaller tumors than mock shRNA-infected cells (shCont. Figure 2D). Immunohistochemistry was performed using hematoxylin-eosin and Ki-67. Along with the knockdown of *CFC1*, the extracellular matrix increased and the percentage of Ki-67-positive cells significantly decreased. The knockdown of *CFC1* in primary NB spheres was also performed using NB1 cells. Concordantly, the knockdown of *CFC1* suppressed sphere formation in primary NB cells and cell line experiments (Figure 2E).

### CFC1 accelerates NB cell aggressiveness

**Figure 3:**
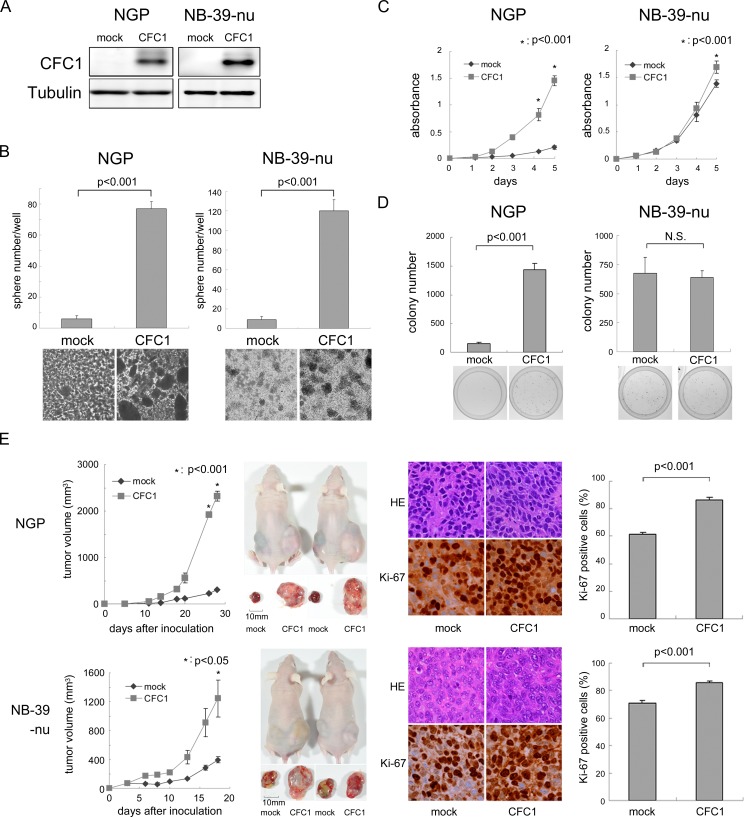
CFC1 accelerates NB tumor formation and aggressiveness **A**. *CFC1* transduction by lentivirus infection. NGP and NB-39-nu cells were infected with a mock or *CFC1*-expressing lentivirus. A Western blot analysis of CFC1 was performed. Tubulin was used as a loading control. **B**. After a 10-day culture in SFM, spheres were counted under a microscope. **C**., **D**. The WST-8 assay and soft agar colony formation assay were performed using *CFC1*-expressing NB cells. Data are representative results of five (C) and four (D) independent experiments. **E**. Tumor development in BALB/c AJcl nu/nu mice following an injection of mock (mock) and *CFC1*-expressing (CFC1) NB cells. Tumor volumes were measured every 2 or 3 days. Data are presented as the mean ± SE of tumors in four mice. Immunohistochemistry was performed using hematoxylin-eosin and Ki-67. The % of Ki-67-positive cells was counted. Data are shown as the percentages of Ki-67-positive cells/field (mean ± SD) in at least three independent fields. Statistical analyses were performed using the Student's *t-*test. The original magnification of each panel is ×400.

We overexpressed *CFC1* in NGP and NB-39-nu cells (Figure 3A). NGP and NB-39-nu cells formed very few tumor spheres under SFM culture conditions. In these two cell lines, sphere numbers and sizes were markedly increased by the overexpression of *CFC1* after 10 days in the SFM culture (Figure 3B). The WST assay was performed using these cells in an adherent culture. CFC1 accelerated the proliferation of cells in both cell lines (Figure 3C). Cell growth was increased under anchorage-independent conditions in NGP cells only (Figure 3D). *CFC1*-expressing NB cells were injected into the subcutaneous regions of nude mice, and formed significantly larger tumors than the mock-infected cells of both cell lines (Figure 3E). Pathologically, the extracellular matrix decreased and the number of Ki-67-positive cells increased with the overexpression of *CFC1*. Collectively, these results indicate that CFC1 accelerates NB cell tumorigenesis and aggressiveness.

### Expression profiling of CFC1-overexpressing NB cells

A Western blot analysis of the phosphorylation of AKT, ERK, JNK, and p38MAPK was performed to elucidate the molecular mechanisms underlying CFC1-related NB tumorigenesis. However, only p38MAPK was phosphorylated in NGP cells overexpressing *CFC1* and the opposite results were obtained for NB-39-nu cells (Supplementary Figure 6).

**Figure 4:**
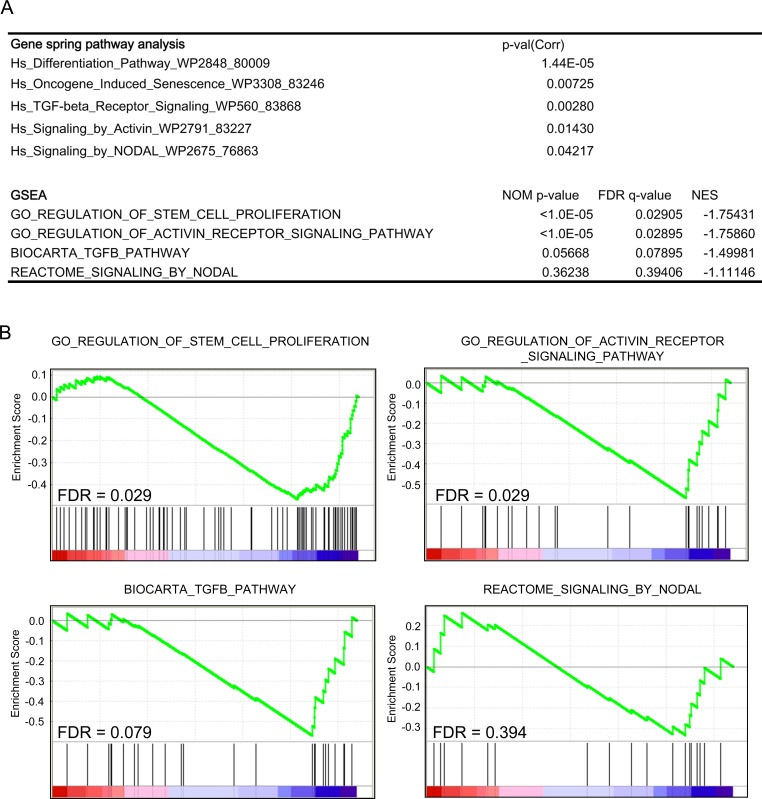
Pathway and gene-set enrichment analysis of *CFC1*-overexpressing cells A transcriptome analysis of *CFC1*-overexpressing NGP cells. A GeneSpring pathway analysis and Gene-set Enrichment Analysis (GSEA) were performed. Collected p-values, nominal p-values, FDR, and normalized enrichment scores (NES) were listed. **A**. Table showing the pathways and gene sets enriched in *CFC1*-overexpressing NGP cells. **B**. The details of GSEA data were displayed with FDR.

We then performed a microarray analysis of *CFC1*-expressing NGP cells to assess its effects on transcriptional profiles. Expression data in *CFC1-* and mock-NGP cells were generated and analyzed using a pathway analysis in GeneSpring GX 13.1 and Gene Set Enrichment Analysis (GSEA) [20]. The pathway analysis was performed for DEGs, defined as the up-regulation of expression by more than 2-fold and a *p*-value in the *t*-test of less than 0.05. Both analyses showed that genes involved in “differentiation”, “oncogene-induced senescence”, “stem cell proliferation”, and “Activin signaling” pathways were significantly enriched in DEGs. The “TGFβ pathway” and “Nodal pathway” were also enriched in the GeneSpring GX pathway analysis, but were not significant in the GSEA analysis (Figure 4, Supplementary Figure 7). This result suggests that the pathways involved in cell stemness and Activin signaling were enhanced by the expression of *CFC1* in NB cells accompanied by transcriptional changes in their related gene members.

### CFC1 suppresses Activin-induced NB cell differentiation

**Figure 5:**
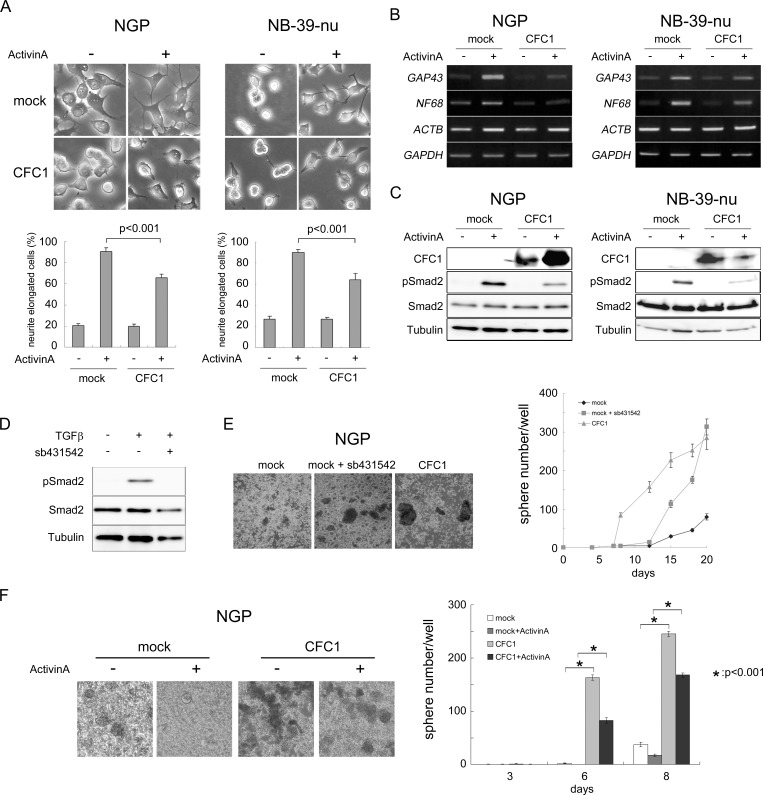
CFC1 inhibits Activin-induced NB cell differentiation and Smad2 activation NGP and NB-39-nu cells were infected with a mock or *CFC1*-expressing lentivirus (CFC1). **A**. Mock and *CFC1*-expressing cells were treated with (+) or without (−) 3 nM of Activin A. Neurite elongation was scored by the presence of neurites longer than one cell in diameter 72 h after the treatment (photo and bar graphs). **B**. The expression of the NB differentiation-related molecules *NF68* and *GAP43* was assessed by RT-PCR. *GAPDH* was used to confirm control *ACTB* expression in the presence of Activin A. C. The protein levels of CFC1, phospho-Smad2, total-Smad2, and tubulin were analyzed by a Western blot analysis. **D**. NGP cells were cultured with 10 μM of the Activin receptor inhibitor sb431542 overnight and treated with 5 ng/ml of TGFβ for 2 h. The level of phosphorylation of Smad2 was assessed by a Western blot analysis. **E**. NGP cells were cultured in SFM with or without sb431542. *CFC1*-overexpressing cells (CFC1) were also cultured as a control. **F**. Mock and *CFC1*-overexpressing NGP cells were treated with 3 nM Activin A. Sphere numbers were counted on days 3, 6, and 8. All data are presented as the mean ± SD of at least three independent experiments. Statistical analyses were performed using the Student's t-test.

Activin A has been shown to induce neurite extension in the NB cell line SK-N-SH-N [21], reduce the malignant phenotype of the NB cell line Kelly, and correlates with a good prognosis in NB patients [22]. The results of the transcriptome analysis with *CFC1*-expressing NGP cells (Figure 4) prompted us to investigate the role of CFC1 in Activin A-induced NB cell differentiation. We cultured mock- or CFC1-transduced NGP and NB-39-nu cells with or without Activin A (3 nM). Neurite elongation was observed after a 72-h culture (Figure 5A). Activin A markedly accelerated neurite extension and CFC1 significantly inhibited the differentiation phenotype. In order to evaluate these effects further, RT-PCR was performed on the neuronal differentiation marker molecules, *GAP43* and *NF68*. Since we expected the expression levels of actin to increase with neurite extension, we used *GAPDH* as a loading control added to *ACTB*. The expression of *GAP43* and *NF68* was up-regulated by Activin A, while CFC1 had the opposite effect (Figure 5B). We then assessed the effects of CFC1 on the Activin A signaling pathway. Since Activin A normally transduces Smad2/3 signaling in neuronal cells [21], a Western blot analysis of Smad2 phosphorylation was performed. The phosphorylation of Smad2 was induced by Activin A and CFC1 reduced the effects of Activin A on Smad2 phosphorylation in both NB cell lines (Figure 5C). In order to examine the role of Activin A signaling in NB tumor sphere formation, we treated NB cells with the Activin receptor inhibitor sb431542. Smad2 phosphorylation was markedly reduced by 10 μM of sb431542 (Figure 5D). Furthermore, tumor sphere experiments indicated that sb431542 significantly increased sphere formation in NGP cells; the expression of CFC1 also had a significant effect on sphere formation (Figure 5E). Mock and *CFC1*-overexpressing NGP cells were treated with 3 nM Activin A. Sphere numbers were counted after 3, 6, and 8 days. Activin A strongly suppressed, whereas CFC1 significantly increased sphere formation, even in the presence of Activin A (Figure 5F). These results suggest that CFC1 suppresses NB cell differentiation and accelerates tumor sphere formation via the Activin A signaling pathway.

## DISCUSSION

### CFC1 expression was associated with NB tumor sphere formation

In NB, a tumor sphere culture in SFM, the culture condition that supports the proliferation and self-renewal of neural crest stem cells, successfully enriched tumor-initiating cells [23]. We cultured bone marrow aspirates from NB patients using these culture conditions and established several NB primary tumor spheres (Supplementary Figure 1). A tumor sphere assay is regarded as a promising alternative to CSC-related experiments. The number and diameters of the tumor spheres formed may be useful for characterizing the cancer stem/progenitor cell population within a population of cultured cancer cells and within *in vivo* tumors [24]. Morozova O et al. previously compared altered gene expression between NB tumor spheres and normal neural crest-like skin-derived precursor cells/tumor tissues [25]. In the present study, we compared altered gene expression between NB tumor spheres and fresh, frozen tumor tissues in order to identify up-regulated genes with tumor sphere formation. We did not use normal neural crest cells as controls, e.g. neural crest-like cells differentiated from iPS cells, in the present study because we focused on cancer stemness-related gene expression. Genes that were up-regulated by more than 2-fold with *p* < 0.05 and whose strong expression was related to an unfavorable prognosis were selected. By combining NB1 and NB2 results, we selected 206 genes and the top 15 ranking genes were listed in Supplementary Table 1. *KISS1R*, *LRRN2*, and *CFC1* were also selected because they are membrane surface-located receptors or co-receptors, taking the development of targeted therapy into consideration. In the three tumor spheres formed from MYCN-amplified NB cell lines, *CFC1* and the positive control *CD133* were markedly increased and a quantitative PCR analysis confirmed the induction of CFC1 (Figure 1).

In order to investigate the relationship between MYCN expression and CFC1-related phenomena, we performed a Kaplan-Meier analysis using CFC1 expression in the *MYCN*-amplified NB patient group and *MYCN*-not amplified NB patient group (Supplementary Figure 5). The strong expression of *CFC1* correlated with an unfavorable prognosis in the *MYCN*-not amplified NB patient group, but not in the *MYCN*-amplified NB patient group (Versteeg 88 cohort and SEQC-498 cohort). A correlation was not observed between the mRNA levels of *MYCN* and *CFC1*. These results suggest that CFC1 is not a downstream target molecule regulated by MYCN.

### CFC1 (Cryptic) oncogenic role in NB

CFC1 (cryptic, HTX2, DTGA2) is a member of the EGF-CFC co-receptor family [26, 27]. A functional analysis of CFC1 using knockout mouse experiments indicated that *CFC1*-null mutants survive until birth and display severe left-right laterality defects, but do not exhibit the phenotypes associated with pre-gastrulation patterning and differentiation [28, 29]. Some cases of prostate cancer and carcinoid tumors indicated the strong expression of CFC1 in the cytoplasm (http://www.proteinatlas.org/ENSG00000136698-CFC1/cancer); however, the exact roles of this molecule in tumorigenesis have not yet been elucidated in detail. Importantly, the double null mutant phenotypes for the EGF-CFC genes *TDGF1* (*Cripto*) and *CFC1*, which encode co-receptors for Nodal, showed that they have partially redundant functions in early mouse development [30], suggesting a collaboration between the two EGF-CFC members in cellular phenomena including tumorigenesis.

In contrast, the role of TDGF1, a molecule of EGF-CFC co-receptors, in embryogenesis and tumorigenesis has been extensively examined [18]. CFC1 and TDGF1 are both members of the EGF-CFC family, and are involved in signaling during embryonic development. These two molecules have an EGF-like domain and CFC domain in the middle of the protein. TDGF1 was initially isolated as a putative oncogene from the human teratocarcinoma cell line NTERA2 [31]. TDGF1 is a stimulator of cell growth and is expressed at high levels in the human colon, stomach, pancreas, breast, ovary, endometrium, cervix, testis, and bladder tumors, but is absent or expressed at low levels in their normal counterpart tissues [32].

We examined the effects of CFC1 on NB tumorigenesis and aggressiveness (Figures 2 and 3). The depletion of *CFC1* by shRNA significantly suppressed tumor sphere formation in 3 NB cell lines, and xenograft tumor formation in IMR32 cells along with Ki-67 positive signals decreased. Furthermore, the overexpression of *CFC1* in 2 NB cell lines up-regulated tumor sphere formation and xenograft tumor formation along with increases in the Ki-67-positive signal, and cell proliferation under normal cell culture conditions and soft agar colony formation were clearly induced in *CFC1*-expressing NGP cells. Taken together, these results indicate CFC1 oncogenic ability, particularly that related to the stemness of NB cells. *CFC1* was detected as one of five DEGs that had high diagnostic accuracy associated with malignant pheochromocytomas by genome-wide expression profiling, suggesting CFC1 oncogenic ability in neural crest-related cell lineages [33]. In primary NB tumors and cell lines, genomic gain or loss in the *CFC1* genomic region 2q21.1 is rare [34, 35]. Furthermore, tumor sphere formation up-regulated *CFC1* transcription in primary tumor spheres and NB cell line spheres (Figure 1), suggesting the epigenetic regulation of *CFC1* in NB cells.

We performed microarrays using *CFC1*-overexpressing NGP cells and found significant transcriptional changes in the gene members for “differentiation”, “oncogene-induced senescence”, “stem cell proliferation”, and “Activin signaling” pathways. Therefore, we investigated Activin signaling in more detail (Figure 5). Of note, the expression of *BMP4*, *TGFB1*, and *TGFB3*, which was previously reported to induce differentiation [36], was down-regulated. The expression levels of *CDK4*, *CDKN2A*, *p14-ARF*, and *p16-INK4A*, a tumor suppressor of NB [37, 38], were decreased in *CFC1*-overexpressing cells. *YAP1* has been described as an oncogene in ovarian cancer [39], and Schramm et al. reported the stronger activation of *YAP1* in relapsed NB than in primary NB [40]. *SMARCD3* was shown to be more strongly expressed in advanced NB than in early stage NB [41]. *NF1* has been described as a tumor suppressor in NB [42]. These findings suggest that CFC1 accelerates tumor malignancy in NB.

### CFC1 suppresses NB differentiation via the Activin pathway

TDGF1 modulates signaling by other TGFβ family ligands, including Activin and TGFβ [43]. The overexpression of TDGF1 antagonizes the Activin-induced growth suppression of tumor cells and accelerates tumor growth in xenograft experiments.

The down-regulation of Activin A has been reported in MYCN-overexpressing NB cells [44]. Previous studies showed that the overexpression of Activin A reduced soft agar colony and xenograft tumor formation, while its weak expression correlated with an unfavorable prognosis in NB patients [22, 45]. Additionally, a Kaplan-Meier analysis using the R2 database indicated that the strong expression of *ACVR2A* correlated with a favorable prognosis (Supplementary Figure 3). Therefore, Activin signal pathways are involved in tumor suppression in many tumors, including NB.

These results prompted us to investigate the effects of CFC1 on Activin A-induced NB cell differentiation. Activin A effectively induced differentiation in the NB-39-nu and NGP cell lines, which was confirmed by neurite extensions and the induction of the neuronal markers *GAP43* and *NF68*. The expression of CFC1 significantly suppressed Activin A-related NB differentiation along with the inhibition of Smad2 phosphorylation (Figure 5). Furthermore, the Activin receptor inhibitor sb431542 suppressed Smad2 phosphorylation and promoted tumor sphere formation in NB cells (Figure 5), suggesting that the Activin signal pathway is involved in NB stemness. This is the first study to clarify the functional role of CFC1 in the Activin signal pathway and suggest the importance of the mechanism suppressing differentiation in NB CSC-model tumor spheres. Although CFC1 has been shown to interact with ACVRIIB and/or GDF1 in *Xenopus* embryos [43], further biochemical analyses are required in order to clarify the exact mechanisms responsible for CFC1-related phenomena in Activin signals.

## MATERIALS AND METHODS

### Primary tumor cells from NB bone marrow aspirates

Bone marrow aspirates from NB patients were sent to the Chiba Cancer Center Research Institute from hospitals around Japan. The present study was approved by the Chiba Cancer Center Ethics Board. Informed consent was obtained at each institution or hospital. We used Lymphoprep (Funakoshi, Tokyo, Japan) to isolate primary tissue samples from bone marrow aspirates according to the manufacturer's instructions. We established several tumor spheres from these samples cultured in SFM, as described below, and 2 of them were examined in this study. Clinical and molecular information was indicated in Supplementary Figure 1A. These samples were free from EB virus infection, which was confirmed by genomic DNA PCR (Supplementary Figure 1B) [46].

### Cell culture of NB cells

Human NB cell lines (IMR32, NGP, SMS-SAN, and NB-39-nu) were obtained from official cell banks (RIKEN Cell Bank, Tsukuba, Japan and ATCC, Manassas, VA, USA) and cultured in RPMI1640 (Wako, Osaka, Japan) supplemented with 10% heat-inactivated fetal bovine serum (FBS, Invitrogen, Carlsbad, CA, USA) and 100 μg/ml penicillin/streptomycin (Sigma-Aldrich, St Louis, MO, USA) at 37°C with 5% CO_2_. In the culture to examine neurite elongation, cells were cultured in RPMI1640 supplemented with 2% FBS and 100 μg/ml penicillin/streptomycin with or without 3 nM Activin A (Sigma-Aldrich).

### Sphere culture of NB cells

The dissociated primary NB cells, IMR32, NGP, SMS-SAN (1×10^5^ per well), and NB-39-nu cells (1×10^4^ per well) were seeded on 6-well plates and cultured in SFM [16]. Half of the medium was replaced with fresh medium every 3 days. After 1-4 weeks of cultivation, the number of spheres was counted under a microscope. In CFC1 overexpression experiments with Activin A, 3 nM Activin A was added to SFM and sphere numbers were counted on days 3, 6, and 8 (Figure 5F). CFC1 knockdown primary spheres (NB1) were loosened by pipetting with an equal volume of AccuMax^®^ (Innova Cell Technologies Inc., San Diego, CA, USA) and seeded on 96-well plates at 250 cells per each well with 100 μL SFM. Eight days later, the number of sphere-positive wells was counted (Figure 2E).

### Semi-quantitative RT-PCR

RNA extraction and a semi-quantitative RT-PCR analysis were performed as described previously [47]. In most cases, *ACTB* was used as a loading control because we expected the expression level of GAPDH to decrease with sphere formation. Primer sequences are described in Supplementary Table 3. RT-PCR results are representative of at least three independent experiments.

### qPCR analysis

We performed a qPCR analysis as described previously [47]. Primers producing 50-150-bp products were designed (Supplementary Table 3). Expression levels were normalized to that of *ACTB*. The results obtained are representative of three independent experiments.

### Microarray analysis

Microarray analyses were performed for primary NB spheres (Affymetrix GeneChip^®^ Human Genome U133 Plus 2.0 Array; Figure 1) and *CFC1*-expressing NGP cells (Agilent Human GE 8×60K v2 design ID 39494; Figure 4). GSE database annotations were 90789 and 90790, respectively. RNA quality was guaranteed by an RNA Integrity Number (RIN) > 6.8. In the primary NB sphere study, transcriptome data were normalized by the mas5.0 algorithm [48, 49]. The definition of DEGs was set as more than 2-fold changes and at least one sample showing the “P” signal.

In the analysis of CFC1-overexpressing NGP cells, 200 ng of total RNA was labeled with Cyanine3 using a Low Input Quick-Amp Labeling Kit (one color, Agilent Technologies) according to the manufacturer's instructions. Purified labeled total RNA was hybridized to SurePrint G3 Human Gene Expression 8×60K Microarray Kit ver2.0 (Agilent Technologies). Hybridization, the scanning of microarrays, and data extraction from scanned images were conducted according to the Agilent protocol version 6.9. A subsequent gene annotation and pathway analysis was conducted using GeneSpringGX 13.1 (Agilent Technologies, Santa Clara, CA, USA).

### Genome-wide survival data analysis

Survival analysis on the data of Obertheur et al. (2014) was conducted by the R2 method (R2: Genomics Analysis and Visualization Platform (http://r2.amc.nl)). The effects of gene expression levels on prognosis. i.e., “high expression predicts poor prognosis” or “low expression predicts poor prognosis,” were determined by the R2's scan method. This method consisted of three steps. First, samples were sorted by expression level. Second, the border was determined to divide the samples into two groups: high and low expression levels. The border was shifted from the top to bottom class to calculate log-rank p-values for all sets. This process was repeated to the last set. Finally, sample groups with the lowest p-value were selected. The p-values were corrected by the Bonferroni method.

### Relationship between CFC1 and MYCN

The prognosis of NB patients was examined using the R2 database (Supplementary Figure 5). Kaplan-Meier curves were drawn according to the expression of *CFC1* in patients with or without the amplification of *MYCN*. CFC1 expression levels were also analyzed in the two groups.

### Knockdown of *CFC1*

pLKO.1-puromycin-based lentiviral vectors containing five sequence-verified shRNAs targeting human CFC1 (RefSeqNM_032545.3) were obtained from the MISSION TRC (Human) shRNA library (Sigma-Aldrich). We transduced these shRNAs using a lentiviral system (described in the next paragraph). We selected two (sh118102: sh1, sh118104: sh2) out of the five shRNAs based on CFC1 knockdown efficiency (data not shown).

### Lentivirus-mediated gene transduction and knockdown

The method used for lentivirus-mediated gene transduction was described previously [16]. HEK 293T cells were used as the packaging cell line and transducing vectors containing the gene [pCDH-CMV-MCS-EF1-Puro (Funakoshi)] or shRNA [pLKO.1 (Sigma-Aldrich)], packaging vectors (Sigma-Aldrich), and the FugeneHD transfection reagent (Roche Applied Science, Indianapolis, IN, USA) were used. Puromycin (Invitrogen) selection was performed after transfection. Transduced cells were analyzed by Western blotting and RT-PCR.

### Cloning of *human CFC1* cDNA

*humanCFC1* cDNA (RefSeq NM_032545.3) was obtained from Invitrogen and sub-cloned into a lentivirus vector using the specific primer sets described in Supplementary Table 3.

### Western blot analysis

Cells were lysed in buffer containing 5 mM EDTA, 2 mM Tris-HCl (pH 7.5), 10 mM β-glycerophosphate, 5 μg/ml aprotinin, 2 mM phenylmethylsulfonyl fluoride, 1 mM Na_3_VO_4_, protease inhibitor cocktail (Nacalai Tesque, Kyoto, Japan), and 1% SDS. The method used for the Western blot analysis was reported previously [51]. In order to detect CFC1, we used a CFC1 polyclonal antibody (ab173858, Abcam, UK). Anti-phospho-, total Smad2, Akt, p38, ERK, and JNK (Cell Signaling Technology) as well as anti-tubulin (Lab Vision, Fremont, CA, USA) antibodies were used.

### Cell proliferation and soft agar assay

Cells were seeded on 96-well plates (750 per well) in culture medium containing 10% FBS. Every 24 h, cell viability was assessed by the water-soluble tetrazolium salt (WST-8) assay using Counting kit-8 (Dojindo, Kumamoto, Japan) as described previously [50]. In the soft agar assay, 2×10^3^ each of IMR32, NGP, SMS-SAN, and NB-39-nu cells were seeded on soft agar as described previously [49]. Viable colonies were stained with 0.05 mg/ml MTT.

### Tumor formation in nude mice

The backs of 6-week-old female athymic BALB/c AJcl nu/nu mice (CLEA Japan, Shizuoka, Japan) were subcutaneously injected with 1×10^6^ IMR32 cells, 5×10^4^ NGP cells, and 1×10^6^ NB-39-nu cells in the presence of 50% (V/V) Corning^®^ Matrigel^®^ as described previously [51]. Tumor sizes were measured every 2 or 3 days. The handling of animals was in accordance with the guidelines of the Saitama Cancer Center Research Institute.

## SUPPLEMENTARY MATERIALS FIGURES AND TABLES









## References

[1] Kreso A, Dick JE (2014). Evolution of the Cancer Stem Cell Model. Cell Stem Cell.

[2] Takeishi S, Nakayama KI (2016). To wake up cancer stem cells, or to let them sleep, that is the question. Cancer Sci.

[3] Tomita H, Tanaka K, Tanaka T, Hara A (2016). Aldehyde dehydrogenase 1A1 in stem cells and cancer. Oncotarget.

[4] Gehling PG, Fargeas CA, Dittfeld C, Garbe Y, Alison MR, Corbeil D, Kunz-Schughart LA (2013). CD133 as a biomarker for putative cancer stem cells in solid tumours: limitations, problems and challenges. J Pathol.

[5] Yiming L, Yunshan G, Bo M, Yu Z, Tao W, Gengfang L, Dexian F, Shiqian C, Jianli J, Juan T, Zhinan C (2015). CD133 Overexpression Correlates With Clinicopathological Features of Gastric Cancer Patients and Its Impact on Survival: A Systematic Review and Meta-Analysis. Oncotarget.

[6] Wang J, Sakariassen PØ, Tsinkalovsky O, Immervoll H, Boe SO, Svendsen A, Prestegarden L, Rosland G, Thorsen F, Stuhr L, Molven A, Bjerkvig R, Enger PØ (2008). CD133 negative glioma cells form tumors in nude rats and give rise to CD133 positive cells. Int J Cancer.

[7] Ricardo S, Vieira AF, Gerhard R, Leitao D, Pinto R, Cameselle-Teijeiro JF, Milanezi F, Schmitt F, Paredes J (2011). Breast cancer stem cell markers CD44, CD24 and ALDH1: expression distribution within intrinsic molecular subtypes. J Clin Pathol.

[8] Croker AK, Allan AL (2012). Inhibition of aldehyde dehydrogenase (ALDH) activity reduces chemotherapy and radiation resistance of stem-like ALDHhiCD44+ human breast cancer cells. Breast Cancer Res Treat.

[9] Liu JC, Deng T, Lehal RS, Kim J, Zacksenhaus E (2007). Identification of tumorsphere- and tumor-initiating cells in HER2/Neu-induced mammary tumors. Cancer Res.

[10] Lo PK, Kanojia D, Liu X, Singh UP, Berger FG, Wang Q, Chen H (2012). CD49f and CD61 identify Her2/neu-induced mammary tumor-initiating cells that are potentially derived from luminal progenitors and maintained by the integrin-TGFβ signaling. Oncogene.

[11] Chen R, Nishimura MC, Bumbaca SM, Kharbanda S, Forrest WF, Kasman IM, Greve JM, Soriano RH, Gilmour LL, Rivers CS, Modrusan Z, Nacu S, Guerrero S (2010). A hierarchy of self-renewing tumor-initiating cell types in glioblastoma. Cancer Cell.

[12] Brodeur GM (2003). Neuroblastoma: biological insights into a clinical enigma. Nat Rev Cancer.

[13] Maris JM, Hogarty MD, Bagatell R, Cohn SL (2007). Neuroblastoma. Lancet.

[14] Hansford LM, McKee AE, Zhang L, George RE, Gerstle JT, Thorner PS, Smith KM, Look AT, Yeger H, Miller FD, Irwin MS, Thiele CJ, Kaplan DR (2007). Neuroblastoma cells isolated from bone marrow metastases contain a naturally enriched tumor-initiating cell. Cancer Res.

[15] Toma JG, Akhavan M, Fernandes KJ, Barnabé-Heider F, Sadikot A, Kaplan DR, Miller FD (2001). Isolation of multipotent adult stem cells from the dermis of mammalian skin. Nat Cell Biol.

[16] Takenobu H, Shimozato O, Nakamura T, Ochiai H, Yamaguchi Y, Ohira M, Nakagawara A, Kamijo T (2011). CD133 suppresses neuroblastoma cell differentiation via signal pathway modification. Oncogene.

[17] Oberthuer A, Juraeva D, Hero B, Volland R, Sterz C, Schmidt R, Faldum A, Kahlert Y, Engesser A, Asgharzadeh S, Seeger R, Ohira M, Nakagawara A (2015). Revised Risk Estimation and Treatment Stratification of Low- and Intermediate-Risk Neuroblastoma Patients by Integrating Clinical and Molecular Prognostic Markers. Clin Can Res.

[18] Rangel MC, Karasawa H, Castro NP, Nagaoka T, Salomon DS, Bianco C (2012). Role of Cripto-1 during epithelial-to-mesenchymal transition in development and cancer. Am J Pathol.

[19] Gray PC, Vale W (2012). Cripto/GRP78 modulation of the TGFβ pathway in development and oncogenesis. FEBS Lett.

[20] Subramanian A, Tamayo P, Mootha VK, Mukherjee S, Ebert BL, Gillette MA, Paulovich A, Pomeroy SL, Golub TR, Lander ES, Mesirow JP (2005). Gene set enrichment analysis: a knowledge-based approach for interpreting genome-wide expression profiles. Proc Natl Acad Sci.

[21] Suzuki K, Kobayashi T, Funatsu O, Morita A, Ikekita M (2010). Activin A induced neuronal differentiation and survival via ALK4 in a SMAD-independent manner in a subpopulation of human neuroblastomas. Biochem Biophys Res Commun.

[22] Schramm A, von Schuetz V, Christiansen H, Havers W, Papoutsi M, Wilting J, Schweigerer L (2005). High Activin A-expression in human neuroblastoma: suppression of malignant potential and correlation with favourable clinical outcome. Oncogene.

[23] Hansford LM, McKee AE, Zhang L, George RE, Gerstle JT, Thorner PS, Smith KM, Look AT, Yeger H, Miller FD, Irvin MS, Thiele CJ, Kaplan DR (2007). Neuroblastoma Cells Isolated from Bone Marrow Metastases Contain a Naturally Enriched Tumor-Initiating Cell. Cancer Res.

[24] Liu JC, Deng T, Lehal RS, Kim J, Zacksenhaus E (2007). Identification of tumorsphere- and tumor-initiating cells in HER2/Neu-induced mammary tumors. Cancer Res.

[25] Morozova O, Vojvodic M, Grinshtein N, Hansford LM, Blakely KM, Maslova A, Hirst M, Cezard T, Morin RD, Moore R, Smith KM, Miller F, Taylor P (2010). System-level analysis of neuroblastoma tumor-initiating cells implicates AURKB as a novel drug target for neuroblastoma. Clin Cancer Res.

[26] Goldmuntz E, Bamford R, Karkera JD, dela Cruz J, Roessler E, Muenke M (2002). CFC1 mutations in patients with transposition of the great arteries and double-outlet right ventricle. Am J Hum Genet.

[27] Shen MM, Schier AF (2000). The EGF-CFC gene family in vertebrate development. Trends Genet.

[28] Gaio U, Schweickert A, Fischer A, Garratt AN, Müller T, Ozcelik C, Lankes W, Strehle M, Britsch S, Blum M, Birchmeier C (1999). A role of the cryptic gene in the correct establishment of the left-right axis. Curr Biol.

[29] Yan YT, Gritsman K, Ding J, Burdine RD, Corrales JD, Price SM, Talbot WS, Schier AF, Shen MM (1999). Conserved requirement for EGF-CFC genes in vertebrate left-right axis formation. Genes Dev.

[30] Chu J, Shen MM (2010). Functional redundancy of EGF-CFC genes in epiblast and extraembryonic patterning during early mouse embryogenesis. Dev Biol.

[31] Ciccodicola A, Dono R, Obici S, Simeone A, Zollo M, Persico MG (1989). Molecular characterization of a gene of the ‘EGF family’ expressed in undifferentiated human NTERA2 teratocarcinoma cells. EMBO J.

[32] Saloman DS, Bianco C, Ebert AD, Khan NI, De Santis M, Normanno N, Wechselberger C, Seno M, Williams K, Sanicola M, Foley S, Gullick WJ, Persico G (2000). The EGF-CFC family: novel epidermal growth factor-related proteins in development and cancer. Endocr Relat Cancer.

[33] Suh I, Shibru D, Eisenhofer G, Pacak K, Duh QY, Clark OH, Kebebew E (2009). Candidate genes associated with malignant pheochromocytomas by genome-wide expression profiling. Ann Surg.

[34] Tomioka N, Oba S, Ohira M, Misra A, Fridlyand J, Ishii S, Nakamura Y, Isogai E, Hirata T, Yoshida Y, Todo S, Kaneko Y, Albertson DG (2008). Novel risk stratification of patients with neuroblastoma by genomic signature, which is independent of molecular signature. Oncogene.

[35] Ohira M, Nakagawara A (2010). Global genomic and RNA profiles for novel risk stratification of neuroblastoma. Cancer Sci.

[36] Chen G, Deng C, Li YP (2012). TGF-β and BMP Signaling in Osteoblast Differentiation and Bone Formation. Int J Biol Sci.

[37] Kamijo T, Zindy F, Roussel MF, Quelle DE, Downing JR, Ashmun RA, Grosveld G, Sherr CJ (1997). Tumor Suppression at the Mouse INK4a Locus Mediated by the Alternative Reading Frame Product p19ARF. Cell.

[38] Takita J, Hayashi Y, Nakajima T, Adachi J, Tanaka T, Yamaguchi N, Ogawa Y, Hanada R, Yamamoto K, Yokota J (1998). The p16(CDKN2A) gene is involved in the growth of neuroblastoma cells and its expression is associated with prognosis of neuroblastoma patients. Oncogene.

[39] Shao DD, Xue W, Krall EB, Bhutkar A, Piccioni F, Wang X, Schinzel AC, Sood S, Rosenbluh J, Kim JW, Zwang Y, Roberts TM, Root DE (2014). KRAS and YAP1 converge to regulate EMT and tumor survival. Cell.

[40] Schramm A, Koster J, Assenov Y, Althoff K, Peifer M, Mahlow E, Odersky A, Beisser D, Ernst C, Henssen AG, Stephan H, Schroder C, Heukamp L (2015). Mutational dynamics between primary and relapse neuroblastomas. Nat Genet.

[41] Takita J, Ishii M, Tsutsumi S, Tanaka Y, Kato K, Toyoda Y, Hanada R, Yamamoto K, Hayashi Y, Aburatani H (2004). Gene expression profiling and identification of novel prognostic marker genes in neuroblastoma. Genes Chromosomes Cancer.

[42] Holzel M, Huang S, Koster J, Ora I, Lakeman A, Nijkamp W, Xie J, Callens T, Asgharzadeh S, Seeger RC, Messiaen L, Versteeg R, Bernards R (2010). NF1 is a tumor suppressor in neuroblastoma that determines retinoic acid response and disease outcome. Cell.

[43] Cheng SK, Olale F, Bennett JT, Brivanlou AH, Schier AF (2003). EGF-CFC proteins are essential coreceptors for the TGFβ signals Vg1 and GDF1. Genes Dev.

[44] Breit S, Ashman K, Wilting J, Ro¨ssler J, Hatzi E, Fotsis T, Schweigerer L (2000). The N-myc Oncogene in Human Neuroblastoma Cells: Down-Regulation of an Angiogenesis Inhibitor Identified as Activin A. Cancer Res.

[45] Panopoulou E, Murphy C, Rasmussen H, Bagli E, Rofstad EK, Fotsis T (2005). Activin A suppresses neuroblastoma xenograft tumor growth via antimitotic and antiangiogenic mechanisms. Cancer Res.

[46] Lay ML, Lucas RM, Ratnamohan M, Taylor J, Ponsonby AL, Dwyer DE, Ausimmune Investigator Group (AIG) (2010). Measurement of Epstein-Barr virus DNA load using a novel quantification standard containing two EVB DNA targets and SYBR Green I dye. Virol J.

[47] Kurata K, Yanagisawa R, Ohira M, Kitagawa M, Nakagawara A, Kamijo T (2008). Stress via p53 pathway causes apoptosis by mitochondrial Noxa upregulation in doxorubicin-treated neuro-blastoma cells. Oncogene.

[48] Yakushiji-Kaminatsui N, Kondo T, Endo TA, Koseki Y, Kondo K, Vidal M, Koseki H (2016). RING1 proteins contribute to early proximal-distal specification of the forelimb bud by restricting Meis2 expression. Development.

[49] Hubbell E, Liu WM, Mei R (2002). Robust estimators for expression analysis. Bioinformatics.

[50] Ochiai H, Takenobu H, Nakagawa A, Ymaguchi Y, Kimura M, Ohira M, Okimoto Y, Fujimura Y, Koseki H, Kohno Y, Nakagawara A, Kamijo T (2010). Bmi1 is a MYCN target gene that regulates tumorigenesis through repression of KIF1Bb and TSLC1 in neuroblastoma. Oncogene.

[51] Aoyama M, Ozaki T, Inuzuka H, Tomotsune D, Hirato J, Okamoto Y, Tokita H, Ohira M, Nakagawara A (2005). LMO3 interacts with neuronal transcription factor, HEN2, and acts as an oncogene in neuroblastoma. Cancer Res.

