# Supplementary material for: CFC1 is a cancer stemness-regulating factor in neuroblastoma
Source: Oncotarget. 2017 Jun 13;8(28):45046–59. doi: 10.18632/oncotarget.18464 (PMC5542166; doi:10.18632/oncotarget.18464)
Supplement: Supplementary file 1 [file oncotarget-08-45046-s001.pdf]

A CFC1 is a cancer stemness-regulating factor in neuroblastoma

Supplementary Material

A

Sample Name	Age at diagnosis	Stage	MYCN	Spcimen type
NB1	33m (relapsed at 120m)	4	Amplified	Bone marrow
NB2	31m	4	Amplified	Bone marrow

B

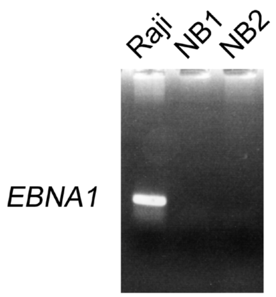

Supplemental Figure 1: Status of NB1 and NB2 cells

(A) Clinical information on NB primary tumors (NB1 and NB2). Primary NB cells were derived from the bone marrow of two patients. NB1 and NB2 were from Stage 4, MYCN-amplified tumors. (B) Status of EB virus infection in primary tumors (NB1 and NB2). Genomic PCR was performed on NB1 and NB2 using specific primers for EBNA1. Primer sequences were described in Supplemental Table 3.

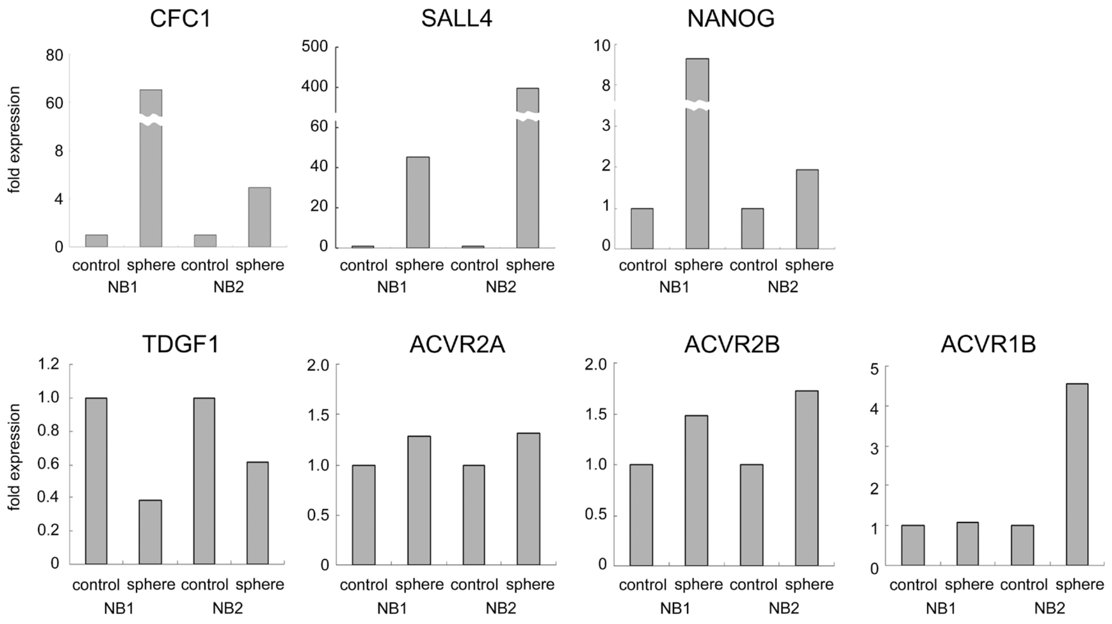

Supplemental Figure 2: Expression of EGF-CFC family pathway-related genes and stemness-related genes in NB tumor sphere

Microarray data on primary NB spheres were generated as described in MATERIALS AND METHODS. The expression of the EGF-CFC family pathway-related genes CFC1, TDGF1, ACVR2A, ACVR2B, and ACVR1C, and stemness-related genes SALL4 and NANOG was assessed. Fold changes from the primary tissue to spheres were shown in bar graphs.

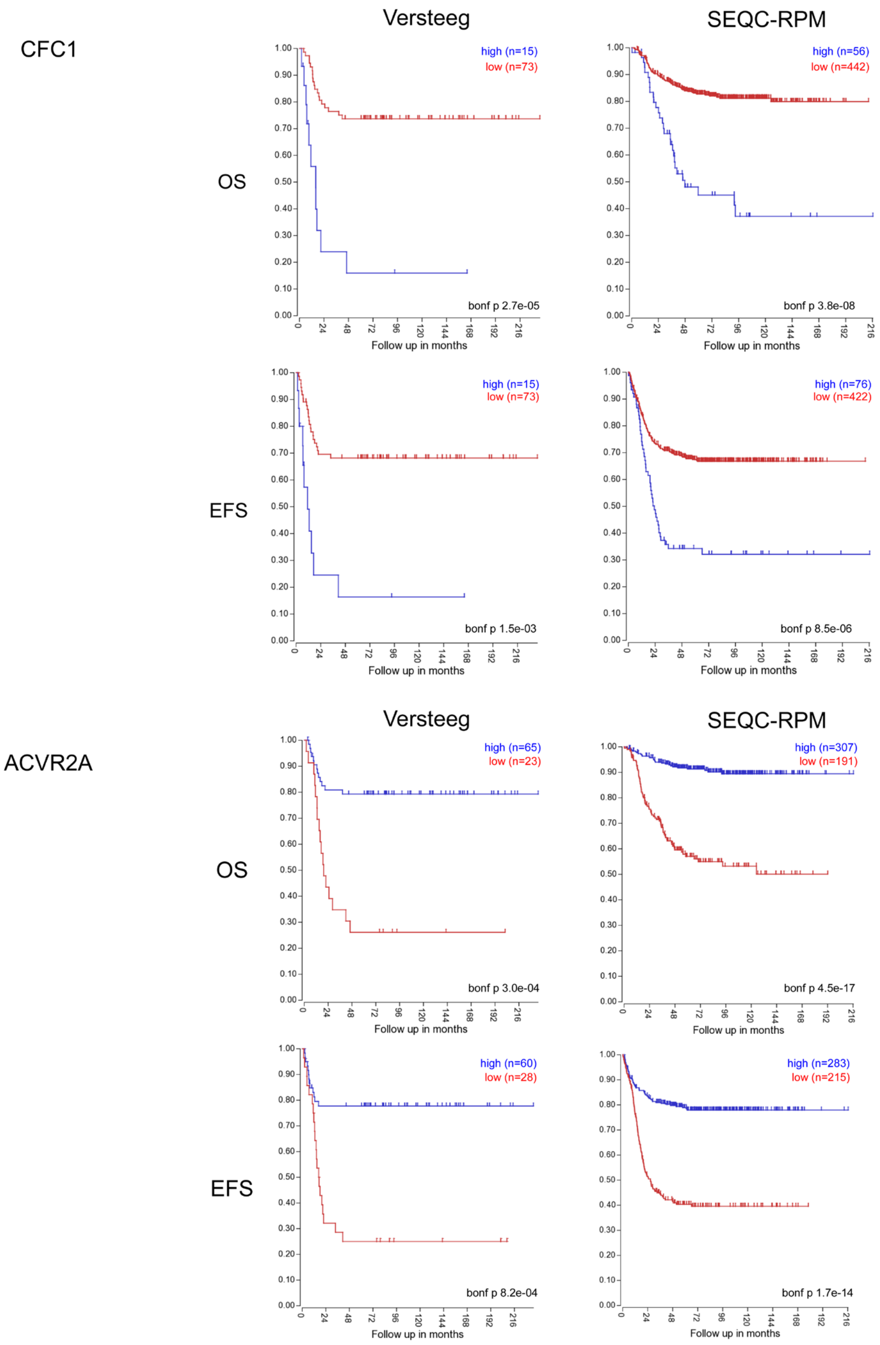

Supplemental Figure 3: Kaplan-Meier curve based on CFC1 and ACVR2A expression

The relationship between EGF-CFC family genes and patient prognoses was described in Supplemental Table 2. The results of CFC1 and ACVR2A, which were significantly different, are shown.

A

B

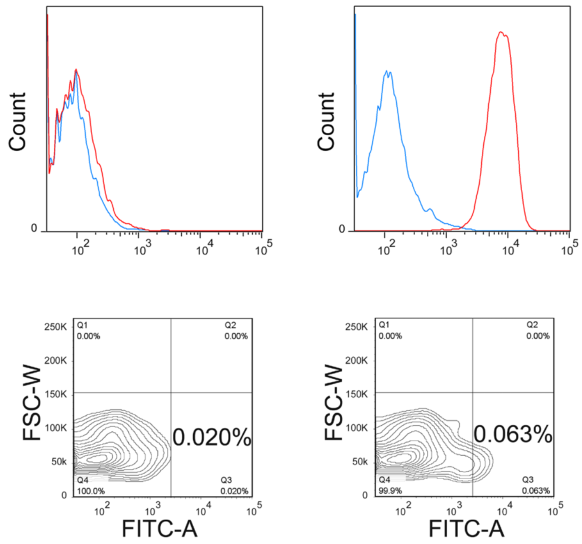

Supplemental Figure 4: Flow cytometry analysis of primary NB cells.
Cells were counted and 1 x 10^6 cells were treated with 0.5 μg of rabbit anti-CFC1 (Abcam, ab17858) or control rabbit-IgG (Wako) on ice for 30 min. Cells were then treated with an Alexa 488-labeled secondary antibody on ice for 30 min, and 0.2 μg/μl of propidium iodide (PI) was added to detect living cells. A flow cytometry analysis was performed using a Canto-II FACS scanner (BD Bioscience) and data were processed with FlowJo software (Digital Biology). (A) Histograms of parental- (left) and CFC1 overexpressing- (right) NGP cells. Cells treated with control-IgG (blue line) or anti-CFC1 (red). (B) Contour plot of primary NB1 cells. Cells were treated with control-IgG (left) or anti-CFC1 (right). Primary NB spheres were loosened with an equal volume of AccuMax®.

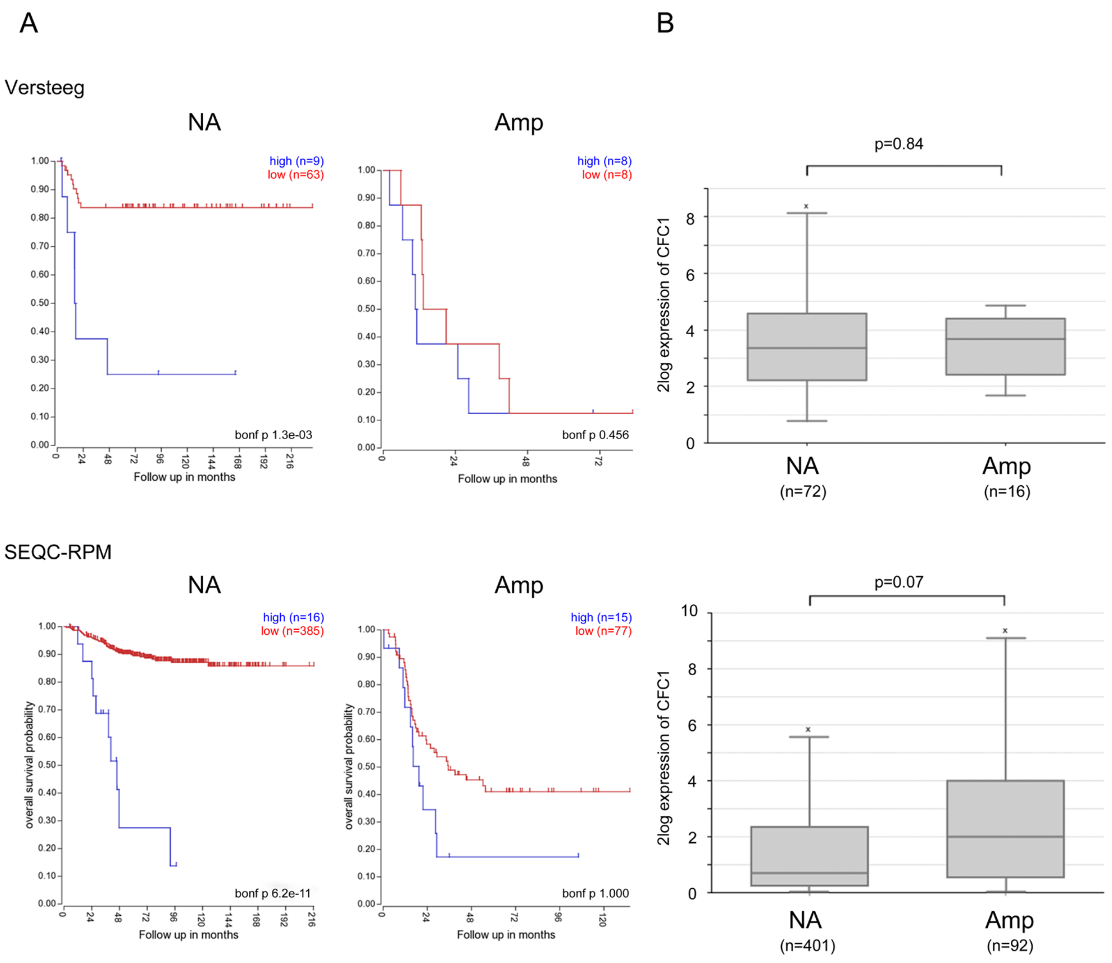

Supplemental Figure 5: Relationship between CFC1 and MYCN expression

The relationship between CFC1 and MYCN expression was examined using the R2 database. Kaplan-Meier curves according to CFC1 expression (A), and CFC1 expression levels were analyzed in tumors with (Amp) or without (NA) the amplification of MYCN (B).

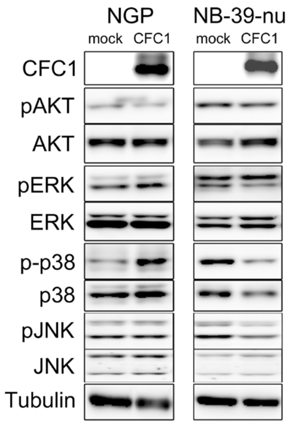

Supplemental Figure 6: Western blot of AKT/ERK/JNK/p38MAPK phosphorylation in CFC1-expressing cells

We performed a Western blot analysis on CFC1-expressing cells. NGP and NB-39-nu cells were infected with mock or CFC1-expressing lentiviruses. The levels of phospho- AKT (p-AKT), total-AKT, phospho-ERK (p-ERK), total-ERK, phospho-JNK(p-JNK), total-JNK, phospho-p38MAPK (p-p38), total-p38MAPK, and tubulin were analyzed using Western blotting.

A

Hs_Differentiation_Pathway_WP2848_
80009 - Homo sapiens

NOG, IL11, PDGFA, WNT2B, ALK, NOTCH1,
PDGFB, NT5E, WNT5A, HGF, SCF, DKK1,
KIT, TGFB3, IL6, TPO, TGFB1, FST, VEGFA, BMP4

Hs_Oncogene_Induced_Senescence_
WP3308_83246 - Homo sapiens

CDK4, CDK6, p16-INK4a, p14-ARF,
CDKN2A, CDKN2C, CDKN2D, ETS1, MDM2

Hs_TGF-beta_Receptor_Signaling_WP560_83868

TGFB1, BMP4, ENG, TGFBR2, FKBP1A, LTBP1,
FST, NOG, ZFYVE9, SMAD2, SMAD3, SMAD6,
SMAD7, HRAS, SPP1, ZFHX1B, RUNX3, FOS

Hs_Signaling_by_Activin_WP2791_83227

ACVR2A, ACVR1C, INHBB, FST, SMAD2, SMAD3

Hs_Signaling_by_NODAL_WP2675_76863

ACVR2A, ACVR1C, GDF1, TDGF1, SMAD2, SMAD3

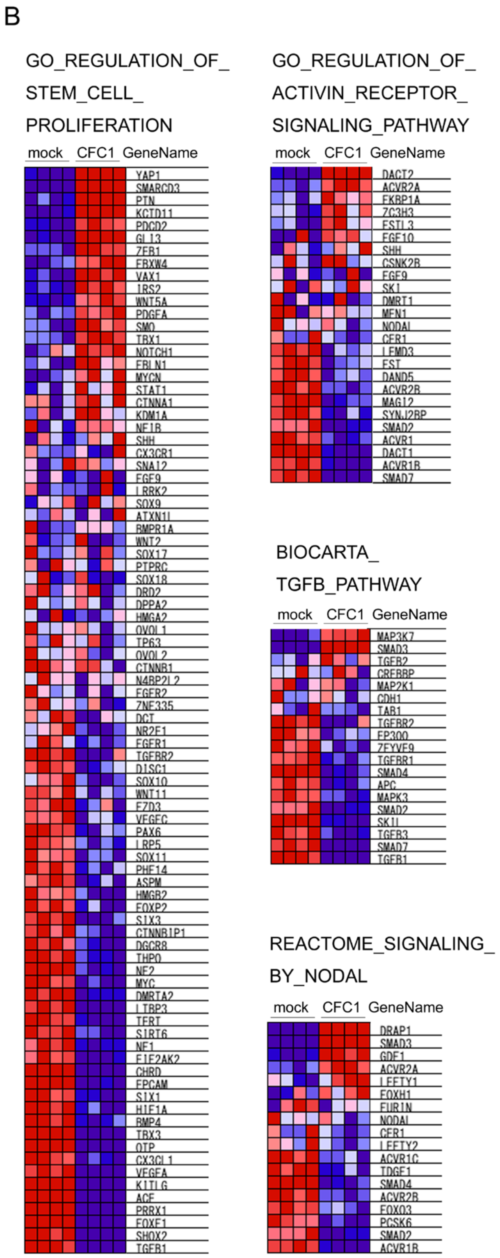

Supplemental Figure 7: Differentially expressed genes in the CFC1 overexpression experiment suggested by Pathway and GSEA analyses. (A) Genes involved in 5 pathways correlated with the overexpression of CFC1 in Figure 4A (GeneSpring Pathway Analysis). (B) Genes involved in 4 pathways correlated with the overexpression of CFC1 in Figure 4A (GSEA analysis). A heat map shows the expression levels of differentially expressed genes indicated by GSEA.

For Supplementary Tables 1, 2,3 see in Supplementary Files
