# Supplementary material for: CFC1 is a cancer stemness-regulating factor in neuroblastoma
Source: Oncotarget. 2017 Jun 13;8(28):45046–59. doi: 10.18632/oncotarget.18464 (PMC5542166; doi:10.18632/oncotarget.18464)
Supplement: Supplementary file 2 [file oncotarget-08-45046-s002.doc]

**Supplementary Table 1: Top 15 genes in the microarray analysis**

Rank Gene Name FC Bonf p GO; components Functions

1 KISS1R 49.973 9.43E-07 cell surface KISS1 receptor

2 ZNF208 21.835 5.76E-05 nucleus zinc finger protein

3 BMX 19.237 4.22E-09 cytosol BMX non-receptor tyrosine kinase

4 KCNJ6 18.721 1.01E-09 Golgi apparatus potassium inwardly-rectifying channel

5 IGF2 14.646 2.50E-07 extracellular space insulin-like growth factor

6 SLC1A6 13.040 7.69E-10 Golgi apparatus high affinity aspartate/glutamate transporter

7 PPP1R3C 12.228 2.04E-51 cytosol protein phosphatase

8 LRRN2 11.468 1.39E-10 membrane leucine rich repeat neuronal

9 SLC30A3 10.809 1.98E-19 lysosomal membrane zinc transporter

10 SPAG4 10.599 2.43E-07 cytoplasm sperm-associated antigen

11 CFC1 10.128 1.53E-05 membrane TGF-beta family co-receptor

12 CLCN7 9.738 1.17E-06 cytoplasmic vesicle chloride channel

13 CNGA3 9.467 3.34E-05 cytoplasm cyclic nucleotide gated channel

14 ASB5 9.385 6.12E-09 intracellular ankyrin repeat and SOCS box containing 5

15 B3GNT7 9.172 8.13E-12 Golgi membrane UDP-GlcNAc:betaGal beta-1,3-N-acetylglucosaminyltransferase 7

FC: Fold change, Bonf p: Bonferroni corrected p-value, GO: Gene Ontology

A microarray analysis was performed on primary NB spheres. The top 15 genes were selected based on fold changes from the primary tissue to spheres. The Gene Ontology Components of these genes were studied.
