# Supplementary material for: CFC1 is a cancer stemness-regulating factor in neuroblastoma
Source: Oncotarget. 2017 Jun 13;8(28):45046–59. doi: 10.18632/oncotarget.18464 (PMC5542166; doi:10.18632/oncotarget.18464)
Supplement: Supplementary file 3 [file oncotarget-08-45046-s003.doc]

**Supplementary Table 2**

OS

Database Versteeg-88 SEQC-498 RPM

Gene Name pCorr unfavorable pCorr unfavorable

*CFC1* 2.70E-05* high 3.80E-08* high

*TDGF1* 0.713 high 0.027* high

*Inhibin bA* 1 high 0.171 low

*Inhibin bB* 0.142 high 1 high

*ACVR1B* 0.554 low 2.00E-15* low

*ACVR2A* 3.00E-04* low 4.50E-17* low

*ACVR2B* 1 low 0.116 low

*TGFB1* 0.041* high 8.90E-10* low

*NODAL* 0.076 low 0.703 low

EFS

Database Versteeg-88 SEQC-498 RPM

Gene Name pCorr unfavorable pCorr unfavorable

*CFC1* 1.50E-03* high 8.50E-06* high

*TDGF1* 1 high 0.02* high

*Inhibin bA* 0.722 low 0.132 high

*Inhibin bB* 1 high 1 high

*ACVR1B* 0.288 low 2.70E-11* low

*ACVR2A* 8.20E-04* low 1.70E-14* low

*ACVR2B* 1 low 9.60E-03* low

*TGFB1* 0.503 high 4.80E-06* low

*NODAL* 1 low 1 high

pCorr: Bonferroni corrected p-value, high: high expression levels correlated with an unfavorable prognosis, low: low expression levels correlated with an unfavorable prognosis

OS: Overall survival, EFS: event-free survival, *: pCorr<0.05

**Supplemental Table 2: Kaplan-Meier analyses according to EGF-CFC family pathway-related gene expression**

Patient prognoses correlated with specific gene expression, e.g. the EGF-CFC family genes *CFC1* and *TDGF1*, and their pathway-related genes were analyzed using the R2 database. P-values (Bonferroni adjusted) for overall survival and event-free survival were listed. Expression cut-off was automatically decided by the R2 algorithm.
