# Supplementary material for: CFC1 is a cancer stemness-regulating factor in neuroblastoma
Source: Oncotarget. 2017 Jun 13;8(28):45046–59. doi: 10.18632/oncotarget.18464 (PMC5542166; doi:10.18632/oncotarget.18464)
Supplement: Supplementary file 4 [file oncotarget-08-45046-s004.doc]

**Supplementary Table 3: Primer information**

Gene Forward Reverse Accession number

*ACTB* CAAGAGATGGCCACGGCTGCT CAAGAGATGGCCACGGCTGCT NM_001101.3

*ACTB* (qPCR) CCTGGCACCCAGCACAAT CCTGGCACCCAGCACAAT NM_001101.3

*CD133* AACTGGCTAAGTACTATCGTCGAATGG AACTGGCTAAGTACTATCGTCGAATGG NM_006017

*CFC1* TCAGAAGCACCGACAGTCAC TCAGAAGCACCGACAGTCAC NM_032545.3

*CFC1* (cloning) AAAATGACCTGGAGGCACCAT TAAAGGCGATGCCCAAGTCC NM_032545.3

*CFC1* (qPCR) TCAGTTTGGCATTACAGATCATCA TCAGTTTGGCATTACAGATCATCA NM_032545.3

*EBNA1* GCCGGTGTGTTCGTATATGG CAAAACCTCAGCAAATATATGAG NC_007605.1

*GAP43* GGAGAAGGCACCACTACTGC GGAGAAGGCACCACTACTGC NM_001130064

*GAPDH* ACCACAGTCCATGCCATCAC ACCACAGTCCATGCCATCAC NM_002046

*KISS1R* CTCGCTGGTCATCTACGTCA CTCGCTGGTCATCTACGTCA NM_032551.4

*LRRN2* CCCGAGACTGTGATTTCCAT CCCGAGACTGTGATTTCCAT NM_006338.2

*NF68*  ACCAAGACCTCCTCAACGTG ACCAAGACCTCCTCAACGTG NM_006158

Primer information for RT-PCR, qPCR, genomic DNA, and sub-cloning used in this study.
